# Knowledge, attitude and behaviour of university students regarding salt and iodine: a multicentre cross-sectional study in six countries in Europe and Asia

**DOI:** 10.1186/s13690-021-00593-5

**Published:** 2021-05-04

**Authors:** Georgios Marakis, Antonios Katsioulis, Lamprini Kontopoulou, Anke Ehlers, Katharina Heimberg, Karen Ildico Hirsch-Ernst, Tomaž Langerholc, Hanna Adamska, Ewa Matyjaszczyk, K. D. Renuka Silva, K. A. Chathurika Madumali, Tai-Sheng Yeh, Ling-Jan Chiou, Mei-Jen Lin, Georgios Karpetas, Anke Weissenborn

**Affiliations:** 1 Nutrition and Food Standards Unit, Directorate of Risk Assessment and Nutrition, Hellenic Food Authority, Athens, Greece; 2 School of Medicine, University of Thessaly, Larissa, Greece; 3 Faculty of Nursing, University of Thessaly, Larissa, Greece; 4 Department of Food Safety, German Federal Institute for Risk Assessment, Berlin, Germany; 5 Department of Microbiology, Biochemistry, Molecular Biology and Biotechnology, University of Maribor, Maribor, Slovenia; 6 Institute of Economic Sciences, Wrocław University of Environmental and Life Sciences, Wrocław, Poland; 7 Institute of Plant Protection-National Research Institute, Poznan, Poland; 8 Department of Applied Nutrition, Wayamba University of Sri Lanka, Gonawila, Kuliyapitiya, Sri Lanka; 9 Department of Food Science and Nutrition, Meiho University, Neipu, Pingtung Taiwan; 10 Department of Health Business Administration, Meiho University, Neipu, Pingtung Taiwan; 11 Department of Animal Science, National Pingtung University of Science and Technology, Neipu, Pingtung Taiwan

**Keywords:** Salt, Iodine, Iodised salt, Europe, Asia, Nutritional epidemiology

## Abstract

**Background:**

Strategies to reduce salt intake are encouraged to be implemented in parallel with those that aim to ensure iodine adequacy at the population level. The aim of the present study was to assess and compare knowledge, attitudes and behaviours related to salt and iodine among students in Europe and Asia.

**Methods:**

A multicentre cross-sectional study was conducted with 2459 university students in total (42.7% males, median age 21 years) from four countries in Europe and two countries in Asia. Data were collected with the use of a self-administered questionnaire, and univariate and multivariate statistical analyses were performed to explore any association between variables.

**Results:**

Only 6.5% of all participants knew the correct salt recommendations. Nearly a quarter of them (24.4%) found salt recommendations confusing and/or contradictory. There were significant differences between European and Asian participants, with those from Europe being better informed about salt recommendations, but significantly less knowledgeable about iodine. The reported frequency of use of salt and salt-containing sauces either at the table or for cooking, as well as knowledge about ways to reduce salt intake among those who indicated to make conscious efforts to do so, differed significantly between countries. Significant differences between countries were also observed with respect to the type of salt used, with about one third of all participants (34%) not being aware of the kind of salt they used.

**Conclusion:**

The results of this survey highlight serious salt- and iodine-related knowledge gaps among university students in Europe and Asia. Raising awareness and conducting information campaigns is needed to promote changes in behaviour that would result in a reduction of salt intake and conscious use of iodised salt at the individual level.

**Supplementary Information:**

The online version contains supplementary material available at 10.1186/s13690-021-00593-5.

## Background

High salt intake is associated with hypertension, which is a recognised major risk factor for cardiovascular diseases. Furthermore, there is evidence that a diet high in salt may also increase the risk of stomach cancer, bone and kidney diseases [1, 2]. The estimated mean population salt intake among adults worldwide is approximately 10 g/day [3], with estimates of more than 12 g/day in many Asian countries [4], which is more than double the maximum amount recommended for adults by the World Health Organisation (WHO), i.e. 5 g/day [5].

Dietary salt reduction has been described as one of the most cost-effective public health policies to reduce the risk of noncommunicable diseases. Therefore, the goal of the WHO and its Member States is to achieve a reduction of the average salt consumption by 30% by 2025 [6, 7]. Raising consumer awareness and improving knowledge through health education programmes constitute a key pillar of salt reduction [8] and may contribute to lower population salt intake [9]. Despite the overwhelming scientific evidence of the link between high dietary salt intake and blood pressure elevation, a limited number of recently published studies dispute the necessity of salt reduction at the population level [10]. Although the methodological pitfalls have been addressed [4, 11], the messages conveyed by these studies tend to confuse the general public. Therefore, consumers’ knowledge, attitude and behaviour (referred to as KAB) related to salt intake need to be routinely assessed in order to create strategic, targeted and evidence-based consumer awareness programmes.

In the area of public health, sharing best practices between countries is a good way to improve performance. While the major contributors to dietary salt intake have previously been reported to differ between countries [12], globalisation in food habits may also result in similar future programmes of salt reduction and global awareness campaigns. Trends in food marketing (such as increasing marketing of specialty salts in some countries) and potential consumer misconceptions need to be monitored frequently so that health authorities and scientific bodies are able to respond appropriately and in a timely manner to emerging public health and nutrition challenges. Although there is no gold standard method for the assessment of KAB related to salt intake [13], a harmonised methodology using the same tool may facilitate the comparison of data between countries [8].

At the international level, salt reduction strategies are encouraged to be implemented together with salt iodisation programmes [14]. Inadequate iodine intake increases the risk of numerous health consequences known as iodine deficiency disorders (IDD). According to the WHO, it is estimated that almost one third of the world’s population has an insufficient iodine intake. Salt iodisation has been introduced in many countries as a cost-effective and sustainable means of preventing or eliminating IDD [15, 16]. However, even in countries with mandatory universal salt iodisation (USI) programmes, certain segments of the population may not attain an adequate iodine intake [17]. Better knowledge regarding the role of iodine in health has been associated with higher dietary iodine intake [18–20], while lack of success of salt iodisation programmes has been partly ascribed to insufficient information and education of the public about iodine and IDD as well as on ways to prevent suboptimal intakes [21]. Raising awareness of salt and iodine should ideally start at a young age, since later in life, changes in behaviours related to nutrition are more difficult to achieve.

The objective of the present study was to examine and compare KAB related to salt and iodine among university students in six countries in Europe and Asia (Germany, Greece, Poland, Slovenia, Sri Lanka and Taiwan) with a high salt intake, different dietary sources of salt and iodine and different types of legislation for salt iodisation.

## Methods

### Participants

A multicentre cross-sectional survey was conducted in university cities of four European countries (Berlin in Germany, Larissa and Thessaloniki in Greece, Wroclaw in Poland and Maribor in Slovenia) and two Asian countries (Kuliyapitiya in Sri Lanka and Pingtung in Taiwan). The students’ data were collected via a self-administered questionnaire. Nutrition/dietetics, medical and veterinary students were excluded a priori, since they are expected to have better knowledge and/or behaviour related to salt and iodine compared to other students [22]. Students that had previously attended lectures on nutrition could not be excluded at the outset, but this information was recorded for a better characterisation of the study population.

The selection of the countries was based primarily on the presence of research collaborators who expressed interest in the study. In each of the participating cities, universities were informed about the objectives of the study. In each country, university departments/classrooms were selected randomly and lecturers asked for permission to conduct the survey before the questionnaires were administered to students. The students, after detailed explanation of the aims and purpose of the survey, were invited to voluntarily and anonymously participate. It was explained that, by answering the questionnaire, participants express implied consent to participate. The estimated time to complete the questionnaire was between 10 and 15 min. Data were collected between June 2018 and April 2019.

The study was approved by the relevant institutional ethical review boards in each country. Data collection was in accordance with the guidelines laid down in the Declaration of Helsinki, and ethical standards of research, especially with respect to the protection of data privacy, were met in all countries.

### Questionnaire

The questionnaire was self-developed and based on previous experience [23]. Prior to commencement of data collection, the questionnaire was circulated among experts (*n* = 5) for comments on both survey content and item construction, and it was pretested for face validity by assessing presentation, phrasing and clarity of instructions. The questionnaire was initially developed in English and then translated into the official languages of the participating countries. To ascertain the accuracy of translation, the questionnaire was initially translated in each country by one person fluent in English and then translated back into English by a different person who was also fluent in English. The questionnaire was tested in a small sample of students to ensure that the items were unambiguous and that the type, flow and number of questions were appropriate. In order to assess the reliability of the questionnaire over time, a small number of students (*n* = 41) in Greece was requested to answer the questionnaire twice over a period of 2 weeks (i.e. test-retest reliability). By use of McNemar’s test and Spearman’s rank correlation coefficient, no statistically significant differences were identified between the responses obtained at these two time points.

The first section of the questionnaire focused on demographic characteristics of the participants, including height and weight, which were self-reported and used to calculate the body mass index (BMI). The second part was composed of 19 questions on knowledge related to salt and iodine such as awareness of salt intake recommendations, knowledge of the relationship between high salt or low iodine intake and disease risks, knowledge of main dietary sources of iodine and salt as well as practices and attitudes regarding salt- and iodine-related food purchasing and preparation (See Additional File). The questions were multiple-choice close-ended, except one (i.e. question on the daily salt intake recommendation), which was open-ended to avoid providing hints towards the correct answer. Regarding the latter, the participants were asked to indicate the recommended maximum amount of salt in either grams or teaspoons per day. Since recommendations on salt intake vary slightly between countries and organisations, for the statistical analysis, both 5 g as well as 6 g of salt per day were regarded as correct answers. The questions on relationships between high salt intake and disease risks as well as between insufficient iodine intake and disorders or disease risks included both correct and incorrect answers (to test for possible misconceptions) as well as answers about relationships for which there is not yet sufficient scientific evidence (such as the potential link between high salt intake and obesity).

### Statistical analyses

Calculation of the sample size showed that 400 students per country were required to detect a significant difference of 10% to any dichotomous outcome, using a two-sided test, with a significance level of 0.05 and a power of 80%. Quantitative variables are presented either as mean values with standard deviation or as median values with interquartile range (IQR). Qualitative variables are presented as frequencies with percentages. In the univariate analysis, Chi-square test or Fisher’s exact test was used to identify any association between independent and dependent categorical variables and any difference among countries. Bonferroni correction was used for all pairwise comparisons between countries. The Mann-Whitney test or Kruskal-Wallis test was used to explore differences among groups of categorical variables with regard to quantitative non-normally distributed variables. The multivariate analysis was performed by using a logistic regression analysis for dependent variables that were statistically significant in the univariate analyses, and adjusting for the independent variables.

Gender, country (or continent) of origin, prior nutrition lectures and BMI categories were used as independent variables and knowledge, attitudes and behaviours related to salt and iodine as dependent variables. *P* values of less than 0.05 were considered as statistically significant. Statistical analysis was conducted using IBM SPSS Statistics software version 22.0.

## Results

### Demographic characteristics

The rate of participation was high, i.e. 97% in Germany, 89% in Greece, 98% in Poland, 100% in Slovenia, 100% in Sri Lanka and 80% in Taiwan, and the total number of participants was 2459. The number of participants and the demographic characteristics of the study population are summarised by country in Table 1. As shown in the table, the study samples differed between countries with respect to gender, BMI and prior participation in nutrition lectures. Females were over-represented in the Slovenian sample while males were over-represented in the German sample. While in Slovenia, about half of the participants had attended nutrition lectures in the past, the proportion of those who had attended such lectures was smaller in the other countries (6.5–28.2%). In addition, 23.9% of all participants were classified as overweight or obese, i.e. having a BMI ≥ 25 or ≥ 30, respectively, with the lowest rate in Sri Lanka (17.5%) and the highest in Slovenia (29.3%) (Table 1).

**Table 1:** Demographic characteristics of the samples in participating countries ^i)^

Country	No of participants	Gender [% male]	Age [median (IQR)]	ΒΜΙ ≥ 25 [%]	Nutrition lectures [%]	Interested in nutrition [%]
Germany	371	65.0^a^	22.0 (20.0–24.0)^a,b^	20.0^a,d,e^	12.2^a^	82.5^a,b^
Greece	403	49.4^b^	21.0 (20.0–21.0)^c^	26.8^a,b^	6.5^a^	75.2^a^
Poland	484	50.0^b^	21.0 (20.0–23.0)^d^	22.5^a,d,e^	9.7^a^	85.5^b^
Slovenia	400	28.0^c^	22.0 (20.0–24.0)^a^	29.3^a,c^	50.5^b^	85.0^b,c^
Sri Lanka	400	43.8^b^	22.0 (21.0–23.0)^b^	17.5^d^	21.8^c^	87.0^b,d^
Taiwan	401	47.6^b^	19.0 (18.0–21.0)^e^	27.4^b,c,e^	28.2^c^	60.3^e^
Total	2459	42.7	21.0 (20.0–23.0)	23.9	21.2	79.4

^i)^ Countries with non-significant differences are denoted with the same letter; those with significant differences are denoted with different letters

### Knowledge and attitudes regarding salt and iodine

#### Salt intake recommendations

The participants’ knowledge on salt recommendations as well as on dietary sources of salt and risks for diseases related to high salt intake are presented by country and for the whole study population in Table 2. The percentages of those who indicated to know the maximum daily intake of salt recommended by WHO and national authorities for adults ranged from 5.8% in Sri Lanka to 24% in Slovenia with no statistically significant differences between males and females (*p* = 0.121) or between those with BMIs above or below 25 (*p* = 0.503). However, only about half of those who indicated to know the recommendation were actually able to specify the correct amount, ranging from 2.3% in Sri Lanka to 10.3% in Slovenia. Also, participants from Europe were more knowledgeable about salt recommendations for adults than those from Asia (7.9% vs. 3.6%), even when adjusted for gender, prior nutrition lectures and BMI (AOR = 2.32, 95%CI: 1.53–3.52, *p* < 0.001), and likewise more knowledgeable about the fact that children should consume less salt than adults (72.9% vs. 65.5%) (AOR = 1.44, 95%CI: 1.2–1.74, *p* < 0.001). Furthermore, multivariate analyses adjusted for gender, continent and BMI showed that those with prior attendance of nutrition lectures had better knowledge of salt recommendations for adults (11% versus 5.3%) (AOR = 2.25, 95%CI: 1.59–3.19, *p* < 0.001) but not with regard to recommendations for children (AOR = 1.16, 95%CI: 0.93–1.45, *p* = 0.198). Results also show that a significantly lower percentage of males (64.8%) than females (75.5%) reported correctly that children should consume less salt than adults, even when adjusted for continent, prior nutrition lectures and BMI (AOR = 0.61, 95%CI: 0.51–0.73, *p* < 0.001) (Table 2).

**Table 2:** Participants’ beliefs and knowledge regarding recommendations, dietary sources and disease-risk relationships related to salt ^i)^

	Germany	Greece	Poland	Slovenia	Sri Lanka	Taiwan	Total
Respondents who indicated that they believe to know the amount of salt recommended for adults [% (n)]							
Salt recommendations	17.2 (63)^a^	7.7 (31)^b^	17.1 (83)^a^	24.0 (96)^a^	5.8 (23)^b^	8.5 (34)^b^	13.4 (330)
Respondents who knew the correct answers [% (n)]							
Amount of salt recommended (5–6 g/d)	9.3 (34)^a^	3.7 (15)^b,c^	8.5 (41)^a,b^	10.3 (41)^a^	2.3 (9)^c^	5.0 (20)^a,b,c^	6.5 (160)
Children should consume less salt than adults	75.4 (276)^a^	55.1 (222)^b^	82.6 (398)^a^	76.8 (307)^a^	50.0 (200)^b^	81.0 (325)^a^	70.5 (1728)
Respondents who indicated that the following are the most important sources of salt [% (n)]							
Salt added during cooking	21.4 (74)^a^	31.8 (128)^b,c^	25.5 (123)^a,b^	36.5 (146)^c^	62.2 (248)^d^	48.9 (196)^e^	37.6 (915)
Sauce/stock added during cooking	5.8 (20)^a,c^	5.2 (21)^a,b^	8.1 (39)^a^	1.8 (7)^b^	2.8 (11)^b,c^	4.7 (19)^a,b^	4.8 (117)
Salt added at the table	4.3 (15)^a,d^	18.4 (74)^b^	5.6 (27)^a^	6.3 (25)^a,c^	11.3 (45)^b,c^	1.5 (6)^d^	7.9 (192)
Sauce added at the table	5.5 (19)^a^	3.2 (13)^a,b^	3.1 (15)^a,b^	1.0 (4)^b^	0.5 (2)^b,c^	2.2 (9)^a,b^	2.6 (62)
Salt in processed foods	33.0 (114)^a^	18.4 (74)^b^	9.1 (44)^c^	14.0 (56)^b,c^	1.8 (7)^d^	2.7 (11)^d^	12.6 (306)
Salt naturally occurring in foods	29.9 (103)^a,c^	23.1 (93)^a^	48.7 (235)^b^	40.5 (162)^b^	21.6 (86)^a^	39.9 (160)^b,c^	34.5 (839)
Respondents who indicated that there is a relationship between high salt intake and the following diseases [% (n)]							
Kidney stones	38.6 (141)^a^	52.1 (210)^b^	52.6 (254)^b^	41.0 (164)^a^	33.8 (133)^a^	68.0 (272)^c^	48.0 (1174)
Obesity	24.9 (90)^a^	45.9 (185)^b^	54.1 (262)^b^	49.0 (196)^b^	24.1 (95)^a^	50.5 (202)^b^	42.2 (1030)
Hypertension	59.9 (218)^a^	84.6 (341)^b^	64.4 (311)^a^	60.3 (241)^a^	63.8 (252)^a^	75.5 (302)^c^	68.1 (1665)
Osteoporosis	9.6 (34)^a^	10.9 (44)^a,b^	12.2 (59)^a,b^	12.5 (50)^a,b^	17.5 (69)^b^	27.8 (111)^c^	15.1 (367)
Stomach cancer	11.7 (42)^a^	4.7 (19)^b^	20.3 (98)^c,d^	25.4 (101)^c,e^	14.5 (57)^a,d^	34.5 (138)^e^	18.7 (455)

^i)^ Each country was compared with the other countries. Countries with non-significant differences are denoted with the same letter; those with significant difference are denoted with different letters

**Fig. 1:**
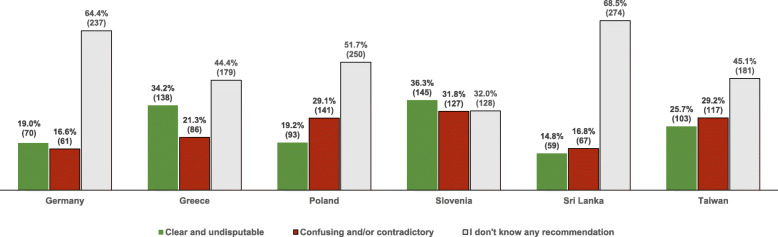
Attitudes of participants towards salt intake recommendations [% (n)]

As depicted in Fig. 1, most participants were either not aware of any recommendations (50.8%) or considered them confusing and/or contradictory (24.4%). Only 14.8 to 36.3% (*p* < 0.001) of the respondents indicated to find the recommendations clear and undisputable.

#### Dietary sources of salt

Concerning the question about the most important sources of salt in the diet, sources reported most frequently by the study group were salt added during cooking followed by salt naturally occurring in foods. Only 12.6% of all participants recognised processed foods as a major source of salt intake, with significant differences between European and Asian students (17.7% vs. 2.3%; *p* < 0.001), also after adjustment for gender, prior nutrition lectures and BMI (AOR = 9.08, 95%CI: 5.59–14.76, *p* < 0.001) (Table 2).

**Fig. 2:**
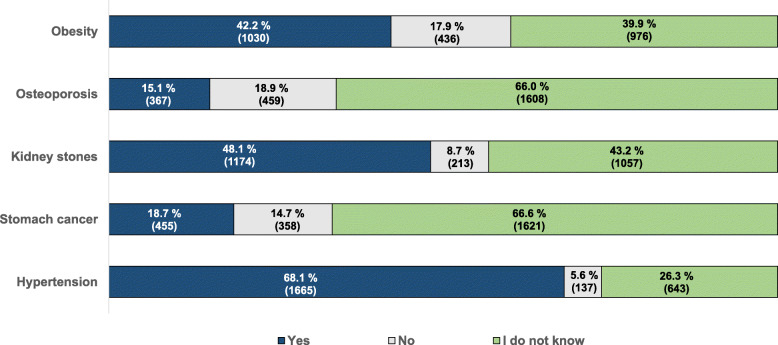
Participants’ responses when asked about the relationship between high salt intake and diseases [% (n)]

Participants’ awareness about the relationship between a diet high in salt and various health conditions was relatively high, irrespective of their origin (Table 2). The proportion of respondents who recognised the relationship between high salt intake and hypertension ranged from 59.9% in Germany to 84.6% in Greece and was overall highest compared with other relationships (Fig. 2), with no significant differences between European and Asian participants (*p* = 0.242). Multivariate analysis, adjusted for continent, previous nutrition lectures and BMI, shows, however, that males (63.6%) were significantly less knowledgeable than females (72.1%) about this relationship (AOR = 0.66, 95%CI: 0.56–0.79, *p* < 0.001) (Table 2).

#### Attitudes towards salt reduction

About two thirds (64.3%) of all participants indicated that salt reduction was important/very important to them. In fact, salt reduction seemed to be important for the majority of the respondents in each of the countries (60.8 to 81%), except for Germany where only one third of the participants (32.8%) indicated that they considered salt reduction as important (Table 3). On the other hand, only a small number of respondents, ranging from 5.2% in Taiwan to 13.2% in Greece, reported making deliberate and conscious efforts to achieve a diet low in salt (Table 3). About two thirds (63.1%) of those who considered salt reduction as not important (overall 35.6%) stated that they were not aware of any recommendations, whereas 20% found the recommendations confusing or contradictory. Multivariate analyses (adjusted for gender, continent, prior nutrition lectures and BMI) show that significantly fewer males (58.1%) than females (69.9%) (AOR = 0.60, 95%CI: 0.51–0.72, *p* < 0.001) believed that salt reduction is important/very important. There were also significant differences in this regard between participants from Europe (58.6%) and Asia (76.1%) (AOR = 0.45, 95%CI: 0.37–0.54, *p* < 0.001) and between participants who had or had not previously attended nutrition lectures (71.9% versus 62.3%; AOR = 1.39, 95%CI: 1.12–1.73, *p* = 0.003) (Table 3).

**Table 3:** Participants’ attitudes towards salt reduction ^i)^

	Germany	Greece	Poland	Slovenia	Sri Lanka	Taiwan	Total
Participants’ attitudes toward salt reduction [% (n)]							
Important or very important^*^	32.8 (120)^a^	60.8 (245)^b^	70.5 (340)^c^	65.8 (263)^b,c^	71.3 (285)^c^	81.0 (323)^d^	64.3 (1576)
Deliberate and conscious efforts for a low salt diet [% (n)]							
Always	7.8 (29)^a,b^	13.2 (53)^a^	8.9 (43)^a,b^	10.3 (41)^a,b^	6.5 (26)^b^	5.2 (21)^b,c^	8.7 (213)
Sometimes	40.8 (151)^a^	39.2 (158)^a^	45.1 (218)^a,b^	52.0 (208)^b^	47.4 (189)^a,b^	40.1 (161)^a^	44.2 (1085)
Never	51.4 (190)^a^	47.6 (192)^a,b^	46.0 (222)^a,b^	37.8 (151)^b^	46.1 (184)^a,b^	54.6 (219)^a^	47.1 (1158)

^i)^ Countries with non-significant differences are denoted with the same letter; those with significant differences are denoted with different letters

^*^Answers 3 and 4 (of the 4-point Likert scale) were combined

When asked to indicate the perception of the saltiness of foods/meals served out of home (restaurants, canteens, fast-food outlets etc.), 52.6% of the respondents from Greece, 45.6% from Taiwan, and 42.8% from Slovenia indicated that they perceived them to be usually saltier than desired, while this was the case in only about a quarter of the respondents, i.e. 26.2, 24.2 and 23.8%, respectively, from Poland, Germany and Sri Lanka (detailed data not presented). Overall, significantly more females than males indicated that they perceived foods served out of home usually saltier than desired, even when adjusted for continent, prior nutrition lectures and BMI (AOR = 0.72, 95%CI: 0.61–0.86, *p* < 0.001). However, there were no significant differences between participants from Europe (36.2%) and Asia (34.8%) in this respect (*p* = 0.468).

#### Knowledge about iodine

As shown in Table 4, depending on their country of origin, participants differed significantly (*p* < 0.001) in their knowledge about the most important dietary sources of iodine. It is noteworthy that 23.7% of the overall study group could not identify any food item of those listed in the questionnaire as a good dietary source of iodine. Interestingly, significantly more males (45%) than females (40.4%) considered iodised salt as a good dietary source of iodine, even after adjusting for country, prior nutrition lectures and BMI (AOR = 1.26, 95%CI: 1.06–1.49, *p* = 0.007). Moreover, after adjustment for gender, prior nutrition lectures and BMI, significantly fewer students from European (34.4%) than from Asian (59.6%) countries regarded iodised salt as an important source of iodine (AOR = 0.36, 95%CI: 0.30–0.43, *p* < 0.001) (Table 4). In addition, only a few of the participants correctly knew that Himalayan salt is a poorer source of iodine than iodised salt (31.9% in Germany, 8.4% in Greece, 1.9% in Poland, 9.3% in Slovenia and 9.2% in Taiwan), while in Sri Lanka, participants were not aware of Himalayan salt at all (detailed data not presented).

**Table 4:** Participants’ knowledge regarding iodine sources, population groups at risk of iodine deficiency and diet-disease relationships ^i)^

	Germany	Greece	Poland	Slovenia	Sri Lanka	Taiwan	Total
Respondents who considered the foods listed as important sources of iodine [% (n)]							
Iodised salt	42.9 (159)^a^	32.3 (130)^b^	32.2 (156)^b^	31.3 (125)^b^	64.5 (258)^c^	54.6 (219)^c^	42.6 (1047)
Fish	59.3 (220)^a,b^	49.9 (201)^a^	60.5 (293)^b^	56.7 (223)^a,b^	38.8 (155)^c^	29.7 (119)^c^	49.4 (1211)
Milk	3.0 (11)^a,c^	4.0 (16)^a,c,d^	1.2 (6)^a^	9.5 (38)^b^	5.8 (23)^b,c^	8.2 (33)^b,d^	5.2 (127)
Seaweed	49.3 (183)^a^	16.9 (68)^b^	26.2 (127)^c^	25.0 (100)^b,c^	32.0 (128)^c^	61.1 (245)^d^	34.6 (851)
Soya sauce	15.6 (58)^a^	6.5 (26)^b,c^	4.5 (22)^b^	7.0 (28)^b,c^	11.0 (44)^a,c^	17.7 (71)^a^	10.1 (249)
Do not know	23.2 (86)^a,d^	34.0 (137)^b^	28.7 (139)^a,b^	29.0 (116)^a,b^	11.0 (44)^c^	15.2 (61)^c,d^	23.7 (583)
Respondents who considered the following population groups at risk of deficiency/requiring additional iodine [% (n)]							
Athletes	49.6 (184)^a^	17.4 (70)^b^	35.7 (173)^c^	37.3 (149)^c^	51.3 (205)^a^	51.9 (208)^a^	40.2 (989)
Elderly	26.7 (99)^a,c^	5.7 (23)^b^	21.3 (103)^a^	6.0 (24)^b^	25.3 (101)^a,c^	30.4 (122)^c^	19.2 (472)
Pregnant	32.9 (122)^a,c^	13.6 (55)^b^	18.0 (87)^b^	12.5 (50)^b^	41.5 (166)^a^	28.7 (115)^c^	24.2 (595)
Lactating	18.6 (69)^a^	9.4 (38)^b^	10.7 (52)^b^	5.8 (23)^b^	20.3 (81)^a^	27.2 (109)^a^	15.1 (372)
Vegetarians	22.1 (82)^a^	9.9 (40)^b^	7.9 (38)^b^	7.0 (28)^b^	13.3 (53)^b^	24.2 (97)^a^	13.7 (338)
Vegans	36.1 (134)^a^	13.4 (54)^b^	13.6 (66)^b^	10.5 (42)^b^	15.8 (63)^b^	34.9 (140)^a^	20.3 (499)
Do not know	25.1 (93)^a^	62.3 (251)^b^	49.4 (239)^c^	51.8 (207)^c^	17.5 (70)^a^	25.2 (101)^a^	39.1 (961)
Respondents who indicated that there is a relationship between low iodine intake and the following conditions [% (n)]							
Obesity	11.7 (40)^a^	14.6 (59)^a^	12.2 (59)^a^	12.5 (50)^a^	22.8 (91)^b^	26.4 (106)^c^	16.7 (405)
Poor cognitive development	18.7 (64)^a,b^	12.2 (49)^a^	24.5 (118)^b,d^	24.8 (99)^b,c,d^	30.8 (123)^d^	47.1 (189)^e^	26.4 (642)
Hypertension	20.4 (70)^a,b^	27.3 (110)^a,d^	21.7 (105)^a,b^	15.8 (63)^b^	43.5174)^c^	36.9 (148)^c,d^	27.6 (670)
Skin rash	12.0 (41)^a,b^	7.4 (30)^a^	12.5 (60)^a,b^	13.3 (53)^a,b^	19.3 (77)^b^	28.4 (114)^c^	15.5 (375)
Thyroid disorders	48.7 (170)^a^	23.6 (95)^b^	44.2 (213)^a^	45.0 (180)^a^	72.2 (288)^c^	70.8 (284)^c^	50.5 (1230)

^i)^ Countries with non-significant differences are denoted with the same letter; those with significant differences are denoted with different letters

Only 24.2 and 15.1% of all participants recognised pregnant and lactating women, respectively, correctly as population groups with higher iodine requirements. Similarly, only 20.3% of the overall study population identified vegans as a risk group for iodine deficiency (Table 4).

Gender and prior knowledge on nutrition were not significantly associated with the ability to correctly identify population groups at risk of iodine deficiency and/or requiring additional iodine (i.e. pregnant and lactating women as well as vegans). However, even after adjustment for gender, prior nutrition lectures and BMI, participants from Europe were significantly less knowledgeable about this than those from Asia, i.e. pregnant women: 18.9% versus 35.1% (AOR = 0.43, 95%CI: 0.36–0.52, *p* < 0.001); lactating women: 11% versus 23.7% (AOR = 0.40, 95%CI: 0.32–0.50, *p* < 0.001); vegans: 17.9% versus 25.3% (AOR = 0.65, 95%CI: 0.53–0.79, *p* < 0.001).

**Fig. 3:**
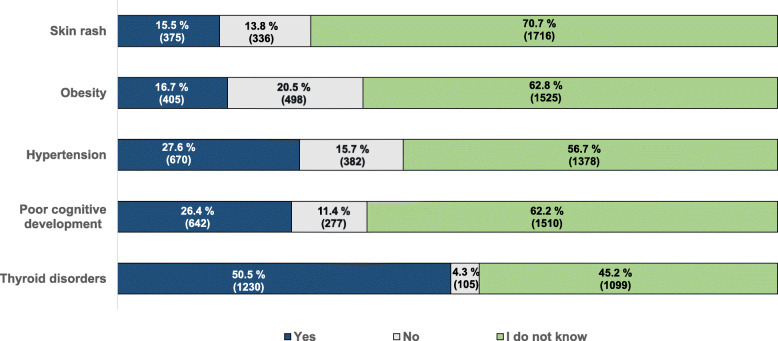
Participants’ responses when asked about the relationship between low iodine intake and diseases [% (n)]

Of the (potential) associations between iodine and disease risks, presented in the questionnaire, half of the overall study group (50.5%) recognised the association between a low iodine status and thyroid disorders correctly, but only 26.4% knew about the association between iodine deficiency and poor cognitive development (Fig. 3) with statistically significant differences between European and Asian participants (20.3% versus 39%) even after adjusting for gender, prior nutrition lectures and BMI (AOR = 0.40, 95%CI: 0.33–0.48, *p* < 0.001). Also, females and those who had attended nutrition lectures in the past were significantly more likely to correctly identify these relationships (*p* < 0.001) (Table 4).

#### Use of salt and sauces at the table and during cooking

Table 5 depicts the types of salt used by the study group in the participating countries. In Greece and Taiwan, almost half of the participants (45.4 and 48.5%, respectively) did not know what type of salt they used. As expected, those who consciously regularly used iodised salt were more likely to consider this as an important source of iodine in the diet (*p* < 0.001). Only 1.4% (0 to 2.5%) of the study group reported not using salt at all.

**Table 5:** Types of salt used by the study population ^i)^

Country	Iodised table salt	Non-iodised table salt	Οther types ^ii)^	Do not know	Do not use salt
	% (n)				
Germany	40.7 (137)^a^	19.3 (65)^a^	21.7 (73)^a,c,e^	17.2 (58)^a^	1.2 (4)^a^
Greece	36.2 (146)^a,b^	2.0 (8)^b^	14.1 (57)^a,b,d^	45.4 (183)^b,d^	2.2 (9)^a^
Poland	28.5 (138)^b,d^	3.9 (19)^b^	26.7 (129)^c^	38.4 (186)^b^	2.5 (12)^a^
Slovenia	42.3 (169)^a^	5.3 (21)^b^	10.3 (41)^d^	41.5 (166)^b,d^	0.8 (3)^a^
Sri Lanka	73.5 (294)^c^	3.3 (13)^b^	13.8 (55)^b,d,e^	9.5 (38)^c^	0.0 (0)^iii)^
Taiwan	21.8 (87)^d^	3.3 (13)^b^	25.3 (101)^c,f^	48.5 (194)^d^	1.3 (5)^a^
Total	40.1 (971)	5.7 (139)	18.8 (456)	34.0 (825)	1.4 (33)

^i)^ Countries with non-significant differences are denoted with the same letter; those with significant differences are denoted with different letters

^ii)^” other types” included salt with added iodine, fluoride and folic acid, rock salt such as Himalayan salt, low sodium salt, sea salt including Fleur de Sel, Piran salt, salt with potassium and magnesium, and pepper salt

^iii)^ This category is not used in comparisons because its column proportion is equal to zero or one

Table 6 presents the behaviour of the study group in terms of salt and savoury sauces used at the table and during cooking. Results show that significantly more European than Asian participants always or more than half of the time added salt and/or sauces at the table (51% versus 27.7%; AOR = 2.64, 95%CI: 2.19–3.17, *p* < 0.001) or during cooking/preparing snacks (75.8% versus 65.4%; AOR = 1.76, 95%CI: 1.44–2.14, *p* < 0.001), adjusted for gender, prior nutrition lectures and BMI. While fewer participants who had attended nutrition lectures than who had not reported frequent addition of salt/sauces at the table (35.8% versus 45.4%; AOR = 0.69, 95%CI: 0.56–0.85, *p* < 0.001), the opposite was observed with regard to adding salt/sauces during cooking (77.9% versus 71.2%; AOR = 1.50, 95%CI: 1.17–1.93, *p* = 0.001), adjusted for gender, continent and BMI. Gender differences were observed only with regard to the habit of adding salt/sauces during cooking, with females being more likely than males to do so (76.3% versus 68.3%, *p* < 0.001). There was, however, no significant difference between those who considered the salt recommendations “confusing and contradictory” and those who found them “clear and undisputable” with regard to their habits of adding salt or sauces at the table (*p* = 0.147) or during cooking (*p* = 0.456), respectively. In addition, participants who considered salt use at the table as the most important dietary source of salt were not more likely to refrain from adding salt at the table than those who did not consider that as an important source (*p* = 0.182).

**Table 6:** Salt-related practices among the study population ^i)^

	Germany	Greece	Poland	Slovenia	Sri Lanka	Taiwan	Total
Respondents who indicated to add always/more than half of the time salt/sauces on the plate [% (n)]							
Salt	47.4 (171)^a,c^	60.8 (245)^b^	55.4 (268)^a,b^	39.0 (156)^c,e^	23.5 (94)^d^	31.9 (128)^d,e^	43.4 (1062)
Soya sauce	6.4 (21)^a^	2.0 (8)^b,c^	5.4 (26)^a,b^	1.8 (7)^c^	-^ii)^	28.5 (114)^d^	7.3 (176)
Other sauces	10.8 (34)^a^	17.4 (70)^a,b^	23.8 (115)^b^	12.0 (48)^a^	-^ii)^	16.2 (65)^a,b^	16.6 (332)
Respondents who indicated to add always/more than half of the time salt/sauces when cooking/preparing snacks [% (n)]							
Salt	90.8 (324)^a^	64.8 (258)^b^	63.8 (306)^b^	89.4 (321)^a,c^	82.4 (285)^c^	49.0 (175)^d^	72.7 (1669)
Stock	10.3 (35)^a^	21.6 (86)^b^	9.8 (47)^a^	20.9 (75)^b^	12.1 (42)^a^	12.3 (44)^a^	14.4 (329)
Soya sauce	8.7 (29)^a^	1.3 (5)^b^	2.9 (14)^b^	1.9 (7)^b^	12.1 (42)^a^	25.2 (90)^c^	8.2 (187)
Other sauces	10.0 (30)^a,b,c^	15.3 (61)^a,b^	16.5 (79)^a^	9.2 (33)^b,c^	12.1 (42)^a,b,c^	7.6 (27)^c^	12.2 (272)
Respondents who reported to check nutrition declaration on food packages for salt content [% (n)]							
Always	3.8 (14)^a,b^	4.5 (18)^a,b^	3.1 (15)^a^	2.3 (9)^a^	5.3 (21)^a,b^	8.7 (35)^b^	4.6 (112)
Sometimes	27.9 (102)^a^	28.8 (116)^a^	33.5 (162)^a^	29.0 (116)^a^	29.3 (117)^a^	47.6 (191)^b^	32.8 (804)
Rarely/never	68.3 (250)^a^	66.7 (269)^a^	63.4 (306)^a^	68.8 (275)^a^	65.4 (261)^a^	43.6 (175)^b^	62.6 (1536)

^i)^ Countries with non-significant differences are denoted with the same letter; those with significant differences are denoted with different letters

^ii)^ This category has not been used for comparisons because its column proportion is equal to zero or one

#### Ways to reduce salt intake

When participants who deliberately aimed at reducing their salt intake were asked to specify ways by which they tried to achieve this, the most popular answers in Greece, Slovenia and Sri Lanka were “by not adding salt/sauce on my plate” (53.1, 56.6 and 36.7%, respectively), while in Poland and Germany, participants most frequently answered “by substituting salt with herbs and spices” (64.4 and 45%, respectively) and in Taiwan “by not adding salt/sauce during cooking” (54.4%). Furthermore, less than half of the respondents indicated “reading food labels and choosing low salt foods” as a means to reduce salt intake (Germany 21.1%, Greece 14.2%, Poland 36.8%, Slovenia 19.3%, Sri Lanka 27.4%, Taiwan 41.2%, *p* < 0.001).

#### Reading of food labels

When asked whether they checked the salt content in the nutrition declaration on food labels, only 3.1 to 8.7% of the participants indicated to always do so (Table 6), while approximately two thirds of the study group (62.6%) indicated to read nutrition declarations rarely or never, except in Taiwan where this applied to only 43.6% (Table 6). Interestingly, those who considered processed foods as the most relevant source of salt in the diet were not more likely to check the nutrition declaration for salt (12.2% always/sometimes versus 12.8% rarely/never; *p* = 0.662). The habit of checking the nutrition declaration on food packages was not statistically different between those who knew or did not know correctly the maximum daily amount of salt recommended (*p* = 0.723). However, participants who were not aware of any salt recommendations (73.1%) or found them confusing and contradictory (53.9%) were significantly more likely to rarely or never check the nutrition information on food labels compared to those (49.8%) who found them clear and undisputable (*p* < 0.001). In addition, multivariate analyses adjusted for gender, continent, prior nutrition lectures, and BMI showed that significantly fewer students from European (33.4%) than Asian (45.5%) countries indicated to always check the nutrition information on food labels (AOR = 0.60, 95%CI: 0.51–0.72, *p* < 0.001), and those with prior attendance of nutrition lectures (43.1%) were significantly more likely (35.9%) to read the nutrition declaration (AOR = 1.29, 95%CI: 1.05–1.58, *p* = 0.013).

## Discussion

This is the first survey that has investigated KAB related to salt and iodine in European and Asian population groups. The results revealed that the majority of participants in all countries were unable to recall or identify the daily maximum amount of salt recommended for adults, which is in line with a recent comprehensive review [24] and other recent studies [25, 26]. Knowledge gaps with regard to the fact that children should consume less salt than adults were also evident in our study group, indicating that despite the self-reported interest of respondents in nutrition or previous attendance of nutrition classes, communication and education about salt-related issues has not been effective in the participating countries. There were only limited data available regarding KAB towards (iodised) salt in the countries included in this survey, particularly in the Asian. However, comparisons of our findings with those from previous studies in Germany [27] and Greece [23] suggest that the knowledge about salt recommendations in these countries has not improved during the past years.

Although national and international public health and scientific organisations consistently recommend salt reduction to about 1 teaspoon (i.e. 5–6 g/day) for adults, a debate has recently been generated by some scientists advocating against this [28], which has been taken up and communicated by journalists to the lay public. Thus, the doubt casted on policies related to salt reduction through social media and other channels of information might have contributed to the fact that nearly a quarter of respondents in our survey found the recommendations regarding salt confusing and/or contradictory.

Reviews on salt-related KAB surveys [12, 24] concluded that consumers are generally able to associate high salt intake with the occurrence of health problems. In those reviews, at least 80% of the respondents were aware that a diet high in salt could lead to high blood pressure. While this was also observed in this study in the majority of participants from Greece, participants from the other countries were less knowledgeable about that. This is worrisome, since according to the Global Burden of Disease Study 2019, high blood pressure appears to be among the three leading risk factors in all participating countries that drive most of the cases of death and disability combined [29]. Consistent with other studies [12, 24], less than 20% of the respondents in this study were able to identify relationships between a diet high in salt and the risks of health conditions such as stomach cancer and osteoporosis, with the exception of kidney stones, which were recognised by almost half of the respondents as being associated with high salt intake. Interestingly, this coincides with the findings of a recent systematic review, which concluded that the evidence regarding the link between salt and blood pressure and kidney disease is better than that for other health outcomes [30]. Moreover, while there is preliminary evidence on the potential role of salt as a risk factor for obesity, through increased consumption of energy-dense food/drinks [31], but also independent of energy intake [32], further evidence is needed to confirm these findings. Interestingly enough, four out of ten participants of our study perceived that a diet high in salt is associated with obesity. This suggests that future studies should investigate various sources of information such as social media and their impact on consumers’ knowledge.

Awareness of the main sources of salt in the diet is important for successful salt reduction. In our study and in agreement with a previous international study [27], Germany was the country with the highest percentage of participants (33% versus 1.8 to 18.4% in the other countries) who indicated correctly that processed foods are the main sources of salt in the diet. Considering that most of the participating countries so far have not achieved significant reductions in the salt content of processed foods [33], raising awareness of the hidden salt in processed foods appears to be timely.

More than 60% of the respondents in all countries, except Germany, considered salt reduction important which is in agreement with results from other international studies [24, 25, 27]. However, while global salt consumption exceeds the recommended maximum levels and many participants considered salt reduction important, only few were making deliberate efforts to reduce their intake, possibly because they underestimated their salt intake, as previously postulated [8]. Furthermore, the relatively young age of the participants as well as the limited knowledge and/or confusion about salt recommendations may also partly explain this attitude.

Reported behaviours to reduce salt intake varied between countries included in this study, reflecting cultural differences as well as divergent messages communicated. The majority of students in this study rarely or never checked the salt content on food labels. This is not surprising when considering the low importance given by most of the participants to the value of checking the salt content on food labels, but also their lack of knowledge about the fact that processed foods are the most important dietary sources of salt and the frequent view that salt recommendations are confusing and/or contradictory. Reading the nutrition declaration on food labels and choosing processed foods with lower salt content has been regarded as one of the essential strategies to reduce salt intake at the population level [34]. A number of front-of-pack labelling systems, which may positively influence food purchasing behaviours, have been developed and are currently being discussed at the international level (e.g. Codex Alimentarius) [35].

Although roughly estimated, the prevalence of discretionary salt use at the table and during cooking in our study was similar to that reported by Grimes and colleagues [36], but higher than that observed in other surveys [8, 12]. In addition, in accordance with other studies [24, 37], in all countries included here, the use of salt for cooking was much more common than the use of salt at the table. In addition, although sauces (such as soya sauce, barbeque sauce, ketchup etc.) or stocks can contribute to salt intake [38], it appeared that the use of table salt, both at the table and for cooking, was more frequent than the use of salt-containing sauces. Considering that processed foods are the main source of salt, the discretionary additional salt use (added at the table or for cooking) becomes worrisome and could be partly attributed to the poor knowledge seen in our study group as well as previously reported by others [39]. This reinforces the notion that knowledge and behaviour are interrelated and that improving knowledge could be an important basis for behavioural changes.

Salt reduction strategies are encouraged to be implemented together with iodisation programmes [15], since iodine deficiency remains a global public health problem despite the progress made in the past decades [18, 40–42]. According to the Iodine Global Network [43], all countries involved in this study appear to have adequate iodine intakes at the population level. However, irrespective of the implementation of USI, certain segments of the populations are considered having or being at risk of an insufficient iodine status for various reasons, such as food choice driven by commercial forces and marketing as well as changes in food consumption habits or by the free trade/single market in the EU [27, 42–49]. For instance, in Poland [50], Slovenia [51] and Sri Lanka [49], mandatory iodisation of salt for household consumption is required by law, whereas in Taiwan [48], Greece and Germany [44], salt iodisation is voluntary and thus both iodised and non-iodised salts are accessible on the market. Indeed, the fact that non-iodised salt may be legally sold in countries of the European Union [52], has been suggested to pose a risk of inadequate iodine intake to those who deliberately choose only non-iodised salt in Slovenia [51]. In Sri Lanka, despite the ban on non-iodised salt, there is still but only a small percentage of households (~ 5%) consuming non-iodised salt.

In Germany, 84% of the population use iodised salt in the household [53], but according to a recent market survey, only about 30% of processed foods contained iodised salt [54]. In the other participating countries, data on the percentage of households consuming iodised salt is scarce. Therefore, strict monitoring along with carefully designed information campaigns and education activities about the importance of iodine for health seem to be of utmost importance for improving and sustaining iodine nutrition.

Food choices made by consumers should be informed and conscious. Studies investigating consumers’ choices and the contribution of iodised salt to the total intake of iodine and salt are limited [40]. In our study, the sample from Sri Lanka had the lowest percentage of respondents who did not know the type of salt they used and the highest reported use of iodised salt. This may be due to better media coverage of the importance of iodised salt through their national salt iodisation programme.

Another aspect of concern is that only less than half of the overall study group regarded iodised salt as an important source of iodine in the diet. Surprisingly, similar findings have been reported elsewhere [21]. Furthermore, a considerable number of participants misconceived Himalayan salt as being a better source of iodine than iodised table salt. Similarly, in a study by Grimes et al. [37], a third of the participants believed that specialty salts are healthier than regular table salt, suggesting that scientifically correct information, which should inform food choices, is not reaching all consumers.

Altogether, results show that further investments in communication and education about iodised salt and IDD are necessary, since lack of knowledge about iodine may be a major risk factor of iodine deficiency [20, 21, 55] while improvement of iodine-related knowledge has been shown to increase sales of iodised salt [56]. In our study, the level of awareness about the benefits of iodine for thyroid function was less than 50% in the participating European countries but approximately 70% in the Asian countries. Poor knowledge of the link between an inadequate iodine status and an impaired cognitive development was also evident and is in line with similar findings by others [19]. While adequate iodine status is vital for the neurocognitive development of the foetus/infant [17], less than half of the respondents in each of the participating countries recognised pregnant and lactating women as risk groups for iodine deficiency. In addition, although vegetarian, but especially vegan diet has been negatively associated with adequate iodine intake [55], less than 40% of the respondents in all participating countries identified vegans as a vulnerable group for IDD. Moreover, the fact that iodised salt was not identified as a major dietary source of iodine shows that knowledge about iodine-rich foods was low in this study group, which is a finding that was also reported in another survey with undergraduate students [21].

Interestingly, respondents from Asian countries appeared to have better knowledge about the relationship between iodine and health than those from Europe. The poor awareness of European populations about iodine has been previously noted [19, 55, 57] and is of concern, since in 2011, the European region was still considered the region with the greatest proportion of children with inadequate iodine intake (43.9%) [17], although in 2015, major parts of Europe were defined as iodine adequate or mildly deficient [43].

While women in our study tended to have better knowledge on the health implications of a diet low in iodine than men, they were not better informed about population groups at risk of iodine deficiency or about those requiring a higher intake of iodine. Low awareness about the importance of iodine among women has been previously reported by others [18, 19, 58] and stresses the need for targeted multi-pronged educational and public health communication strategies.

### Strengths and limitations of the study

The main strengths of this study were the use of the same methodological approach including the procedure followed for the recruitment of participants in all of the participating countries, which resulted in a high response rate with a minimal risk of over-participation of highly health-conscious individuals, and it allowed direct comparisons of results between countries. Although participation rates in all countries were high, it has to be acknowledged that the survey was conducted among university students who are not representative of the whole population. Hence, the outcomes of this survey cannot be generalised or easily extrapolated to the general population in the participating countries. In addition, while trends between European and Asian cohorts were observed in terms of iodine and salt-related knowledge and behaviour, future studies should include more countries from these two continents to draw firm conclusions.

Our survey was based on self-reported information (including self-reported height and weight), which is typically prone to recall and social desirability bias. This might have resulted in misreporting and in conservative estimates of inappropriate dietary behaviours as to the use of additional salt at the table or to reading food labels [8, 9]. However, as the questionnaire was a fully anonymous and self-administered one, the risk of social desirability bias was considered to be low. Finally, there might be differences between self-reported and actual behaviour, which in practice is very difficult to assess [13]. Collection of urine samples could otherwise provide a more realistic picture of the current situation in this population group, both in terms of salt and iodine intakes.

## Conclusion

The results of this survey highlight serious gaps and misinformation regarding salt- and iodine-related knowledge among university students in both developed and developing countries in Europe and Asia. They call for an urgent need for intensive and sustained risk communication and awareness campaigns to promote individual-level behavioural changes. These behavioural changes should include a higher proportion of use of iodised salt in private households and the choice of processed foods that are produced with iodised instead of non-iodised salt as well as a reduction of discretionary general salt intake. Any measures that aim at these changes should be implemented simultaneously in order to spare resources. Raising awareness about salt and iodine recommendations should be intensified in the participating countries and should include better information about the hidden sources of salt in the diet, the importance of reading food labels and the health risks associated with high salt and low iodine intakes, by use of simple, realistic and culturally driven ways of conveying clear and practical messages. All this should be ideally done in parallel with interventions to change the food environment for facilitating healthier choices. It is important that any measures intended to reduce the burden of disease related to excessive salt intake and inadequate iodine supply should start ideally at a young age and be integrated in the curriculum of schools, colleges and universities. Future surveys should also investigate the sources of information that people use and in particular the impact of social media in order to plan targeted consumer education activities.

## Supplementary Information

**Additional file 1.** Questionnaire

## Data Availability

The data supporting the conclusions of this article are available from AK on reasonable request.

## Abbreviations

AOR: Adjusted odds ratio
BMI: Body mass index
CI: Confidence interval
IDD: Iodine deficiency disorders
IQR: Interquartile range
KAB: Knowledge, attitude and behaviour
USI: Universal salt iodisation
WHO: World Health Organization
