# Supplementary material for: Knowledge, attitude and behaviour of university students regarding salt and iodine: a multicentre cross-sectional study in six countries in Europe and Asia
Source: Arch Public Health. 2021 May 4;79:68. doi: 10.1186/s13690-021-00593-5 (PMC8097851; doi:10.1186/s13690-021-00593-5)
Supplement: Supplementary file 1 — Additional file 1. Questionnaire [file 13690_2021_593_MOESM1_ESM.pdf]

Questionnaire on knowledge, attitude and behaviour of university students regarding salt and iodine

Gender: ☐ male ☐ female

Age:

University: ____

Department: ____

If you are an Erasmus or foreign student, please tick here: ☐

Date of questionnaire completion: ______/________/________

Day Month Year

Height: ____________**cm**

Weight: ___________**kg**

Have you attended any courses/lectures on nutrition, while being a student?

☐ yes ☐ no

1. **Are you generally interested in nutrition?**

**Not at all**							**Very interested**
**1**	**2**	**3**	**4**	**5**	**6**	**7**	**8**
☐	☐	☐	☐	☐	☐	☐	☐

2. Do you add salt or sauce to your food on the plate at the table (home and out-of-home)? (tick appropriate box **in each column**)

	**Salt**	**Soya sauce**	**Other types of ready-made sauce/ dressing***
always	☐	☐	☐
More than half of the times	☐	☐	☐
Less than half of the times	☐	☐	☐
Never/ extremely rarely	☐	☐	☐

*** If you have indicated above other types of sauce/dressing, please specify:**

____

____

3. Do you add salt, stock cubes or sauce when cooking or preparing snacks? (tick appropriate box **in each column**)

	**Salt**	**Stock cubes**	**Soya sauce**	**Other types of ready-made sauce/marinade***
always	☐	☐	☐	☐
More than half of the times	☐	☐	☐	☐
Less than half of the times	☐	☐	☐	☐
Never/ extremely rarely	☐	☐	☐	☐

*** If you have indicated above other types of sauce/marinade, please specify:**

____

☐ **I never cook or prepare food/meals**

4. Is the salt you are using most of the times: (tick one answer)

☐ I do not know

☐ Non-iodised table salt

☐ Iodised table salt

☐ Low-sodium salt

☐ Rock salt (e.g. Himalayan)

☐ Salt with added fluoride, iodine & folic acid

☐ Other type of salt:

____

☐ I do not use salt

5. Do you know the amount of salt that the experts recommend to be consumed by adults per day?

☐ yes ☐ no

6. If yes, how much is it per day? (either in grams or number of teaspoons)

__________g

or

__________teaspoons

7. Children should consume:

☐ more salt than adults

☐ The same amount of salt as adults

☐ Less salt than adults

☐ I do not know

8. Is salt reduction important for you?

Not at all			Very important
1	2	3	4
☐	☐	☐	☐

9. Do you know if a diet high in salt is related to any of the following diseases:

	Yes	No	Do not know
Kidney stones	☐	☐	☐
Obesity	☐	☐	☐
High blood pressure	☐	☐	☐
Osteoporosis	☐	☐	☐
Stomach cancer	☐	☐	☐

10. Do you find the recommendations by experts regarding salt:

☐ Clear and undisputable

☐ Confusing and/ or contradictory

☐ I do not know any recommendations

11. Are you deliberately and consciously trying to follow a diet low in salt?

☐ Never/extremely rarely **➔ please, go directly to question 13**

☐ sometimes

☐ always

12. If you answered “always” or “sometimes” in question 11, which of the following are you doing? (You may tick more than one)

- ☐ (a) Not purchase salt from supermarkets/shops
- ☐ (b) Not purchase ready-made sauces from supermarkets/shops
- ☐ (c) Remove salt shaker or ready-made sauce (e.g. soy sauce etc.) from table at home
- ☐ (d) Not add salt or ready-made sauce (e.g. soy sauce etc.) on my plate
- ☐ (e) Not add salt or ready-made sauce (e.g. soy sauce etc.) during cooking/preparation of meals
- ☐ (f) Read the labels and buy foods with less or no salt
- ☐ (g) Substitute salt with herbs and spices
- ☐ (h) Rinse thoroughly canned foods in brine before consuming them
- ☐ (i) Other. Please specify:

____

13. Which of the following is the most important source of salt in the diet of adults? (tick one answer)

- ☐ Salt added during cooking
- ☐ Ready-made sauce/stock cubes added during cooking
- ☐ Salt added on the plate
- ☐ Ready-made sauce (e.g. soy sauce etc.) added on the plate
- ☐ Salt in all types of processed foods (e.g. bread, cheese etc.)
- ☐ Salt naturally occurring in foods

14. Do you find the food served out of home (e.g. in restaurants or canteens) usually:

- ☐ Contains less salt for my preference
- ☐ Contains the right amount of salt
- ☐ Contains more salt for my preference
- ☐ I have not thought about it – I do not take care

14. Do you know if Himalayan salt, as far as its iodine content is concerned, is:
(tick one box)

☐ Better sources of iodine than iodised salt
☐ Poorer sources of iodine than iodised salt
☐ Neither better nor worse
☐ I do not know - I have never heard of Himalayan salt

16. Do you check the nutrition information on the food packages for salt content?

☐ Yes, always
☐ Yes, sometimes
☐ Rarely/never

17. Are any of the items below, in your opinion, good sources of iodine in the diet?

(You may tick more than one box)

☐ (a) Fish	☐ (b) Seaweed	☐ (c) Iodised salt
☐ (d) Red meat	☐ (e) Fruits	☐ (f) Soya sauce
☐ (g) Milk	☐ (h) Nuts	☐ (i) I do not know

18. Do you know if any of the following population groups require additional iodine or are at risk of becoming deficient in iodine? (You may tick one or more answers)

☐ Athletes
☐ Elderly people
☐ Pregnant women
☐ Lactating women
☐ Vegetarians
☐ Vegans
☐ I do not know

19. A diet low in iodine increases the risk for/is related to:

	Yes	No	Do not know
Obesity	☐	☐	☐
Poor cognitive development	☐	☐	☐
High blood pressure	☐	☐	☐
Skin rash	☐	☐	☐
Thyroid disorders/ disease	☐	☐	☐

Thank you for your participation!
